# Lifelong cGAS deficiency leads to altered lipid storage and cholesterol homeostasis

**DOI:** 10.1186/s40659-026-00669-y

**Published:** 2026-01-27

**Authors:** Daniela Carrillanca, Ian Riquelme, Matías Mansilla-Jaramillo, Camila Sánchez-Pérez, Andrea Monterroza, Natalia Lepio, Fabián Rojas, Gonzalo I. Cancino, Paola Murgas

**Affiliations:** 1 Facultad de Medicina, Instituto de Inmunología y Parasitología, Universidad Austral de Chile, Valdivia, Chile; 2 Laboratorio de Neurobiología, Facultad de Ciencias Biológicas, Pontificia Universidad Católica de Chile, 8331150 Santiago, Chile; 3 Escuela de Medicina, Facultad de Medicina, Universidad San Sebastián, Lago Panguipulli 1390, 5501842 Puerto Montt, Chile

**Keywords:** cGAS, cGAMP, STING, Cholesterol, Lipid metabolism, Adipose tissue, Aging, Metabolic homeostasis

## Abstract

**Background:**

The complex interaction between the immune system and metabolic homeostasis is becoming recognized, as immune sensors affect key metabolic tissues, including the liver and adipose tissue. The cGAS–cGAMP–STING pathway, previously recognized as a cytosolic DNA-sensing pathway, is currently associated with lipid metabolism in addition to its inflammatory function. Although STING is acknowledged for its connection to cholesterol, the metabolic functions of its upstream component molecules—the DNA sensor cGAS and the resulting product cGAMP—are largely unexplored. We propose that cGAS and cGAMP serve as crucial, previously unidentified regulators of systemic lipid homeostasis throughout the lifetime.

**Results:**

We investigated the long-term metabolic consequences of intrinsic cGAS deficiency, leading to the absence of cGAMP, in male mice fed on a standard chow diet. cGAS knockout (cGASKO) mice demonstrated a consistent increase in body weight across their lifespan, primarily attributed to adipocyte hypertrophy and increased adipose tissue mass. Increased weight correlated with elevated adiposity. This condition was associated with reduced weight-bearing strength, despite unchanged general locomotor activity and food intake during young age. Liver histology revealed modest cellular infiltration and absent steatosis, suggesting potential low-grade inflammation. Circulating triglyceride and glucose levels exhibited transient, age-dependent variations—decreased glucose and increased triglycerides in young age, which stabilized in adult and old ages, reflecting a possible compensatory metabolic adaptation with time. Conversely, total cholesterol levels were consistently and significantly elevated across all age groups, underscoring the importance of the cGAS–cGAMP axis in cholesterol homeostasis throughout life.

**Conclusions:**

Our study identified the cGAS–cGAMP axis as an interesting regulator of cholesterol homeostasis and fat accumulation in aging, independent of STING activation. The persistent deficiency of cGAS and cGAMP leads to lifelong hypercholesterolemia and adipose hypertrophy. This research highlights an unexpected metabolic function of the cGAS–cGAMP pathway and indicates the necessity of evaluating this axis in relation to physiological aging and metabolic disorders.

## Introduction

Recent findings indicate that lipid metabolism is considerably affected by molecules frequently linked with the immune system [1–3]. In metabolically active organs such as adipose tissue and the liver, many immunomodulatory compounds are prominently produced and are essential for the regulation of immune responses and systemic lipid homeostasis [3, 4]. This intricate connection reflects the growing concept of immunity as a fundamental element of metabolic regulation.

The cGAS-STING signaling pathway has recently demonstrated significant involvement in lipid metabolism [5]. Cyclic GMP-AMP synthase (cGAS) functions as a principal intracellular sensor of double-stranded DNA (dsDNA), detecting both exogenous dsDNA from pathogens and endogenous dsDNA produced from damaged nuclei or mitochondria (mtDNA) [5–7]. Upon the cytoplasmic detection of dsDNA, cGAS facilitates the production of the second messenger 2′-3′ cyclic GMP-AMP (cGAMP) from ATP and GTP [5–7]. cGAMP subsequently associates with the Stimulator of Interferon Genes (STING) protein in reticulum endoplasmic (ER). This ultimately activates inflammatory transcription factors, leading to a robust activation of inflammatory genes, including type I interferons and other pro-inflammatory molecules [5–7]. In obesity and metabolic disorders, cGAS identifies damaged mtDNA produced by stressed mitochondria, activating the cGAMP-STING axis, which enhances the signaling pathway and exacerbates inflammation and tissue damage [5, 6, 8, 9].

Nevertheless, this pathway additionally operates beyond inflammatory processes, playing direct roles in the regulation of metabolism [5, 8–10]. STING, the most well characterized component in this context, functions as a cholesterol-binding protein with structural domains that determine its location within the ER and Golgi [11]. Elevated cholesterol levels inhibit the translocation of STING from the ER to the Golgi and interfere with signaling, thereby establishing a direct connection between lipid availability and immune function [11]. STING additionally interacts with lysosomal cholesterol transport proteins [12] and influences the regulation of lipid-associated genes [8–14]. Despite growing data, the precise role of cGAS and cGAMP—the upstream components of the pathway—in systemic metabolic control during natural aging remains predominantly unexplored.

There is limited data concerning the independent functions of cGAS and cGAMP within this lipidic regulation. The role of cGAMP is poorly understood, as it has constantly been associated with STING activation in the context of metabolic disorders [15]. Notably, cGAS exhibits functional versatility; it can initiate autophagy through interactions with critical regulators [8, 14, 16] and its nuclear location can influence gene expression and β-cell proliferation in diabetes models, regardless of cGAMP synthesis [17, 18]. Moreover, the cGAS-STING pathway has been significantly associated with sterile inflammation and aging [19–22]. Prior research indicated that lifelong STING deficiency results in obesity and increased lipid levels [22]. The total absence of cGAS leads to accelerated aging and chronic [20] (in press). To our knowledge, the long-term metabolic effects of the total lack of cGAS-cGAMP under physiological conditions remain unknown.

Considering that cGAMP is synthesized exclusively by cGAS [7, 9, 23], cGAS knockout mice constitute a distinctive model wherein both cGAS activity and cGAMP production are entirely eliminated. Therefore, the aim of this work was to investigate the long-term effects of cGAS impairment on lipid metabolism in mice feed a standard chow diet, with a focus on identifying metabolic dysregulations and defining the particular function of the cGAS-cGAMP axis throughout their lifespan. We hypothesized that the cGAS–cGAMP axis functions as a significant, previously unrecognized regulator of cholesterol and adipose tissue homeostasis over the lifespan.

## Methods

### Experimental animal groups

The C57BL6J (B6) Wild-Type (WT) (cat.#000664) and cGAS Knockout (cGASKO) (B6(C)-Cgas ^tm1d(EUCOMM)Hmgu^/J) (cat.# 026554) mice, both originating from the same B6 background, were obtained from The Jackson Laboratory, USA. The WT mice serve as the physiological controls, while the cGASKO mice constitute the experimental group. In this context, WT animals, along with their tissues or blood samples, will be used as controls for the cGASKO mice, with all comparisons conducted in reference to them. All mice had unrestricted access to water and were fed with Lab Diet 5P00 Prolab RMH 3000 (a standard chow diet). All mice were housed in groups of four per cage within a room maintaining a 12-h light–dark cycle and a temperature determined between 20 and 22 °C. Male WT and cGASKO mice were employed, excluding females, to eliminate data affected by hormonal variables. Considering that the ablation of cGAS directly abolishes cGAMP production while preserving an intact STING signaling pathway, this comparison is methodologically suitable for isolating the specific effects attributable to cGAS deficiency [24]. The animals were classified by age: young (1 to 4 months), adult (10 to 15 months), and old (21 to 24 months) [25, 26]. The Bioethics and Biosafety Committee of Universidad Mayor, IACUC ID 052018, authorized animal maintenance and all procedures performed in this investigation.

### Body weight assessment

**Fig. 1:**
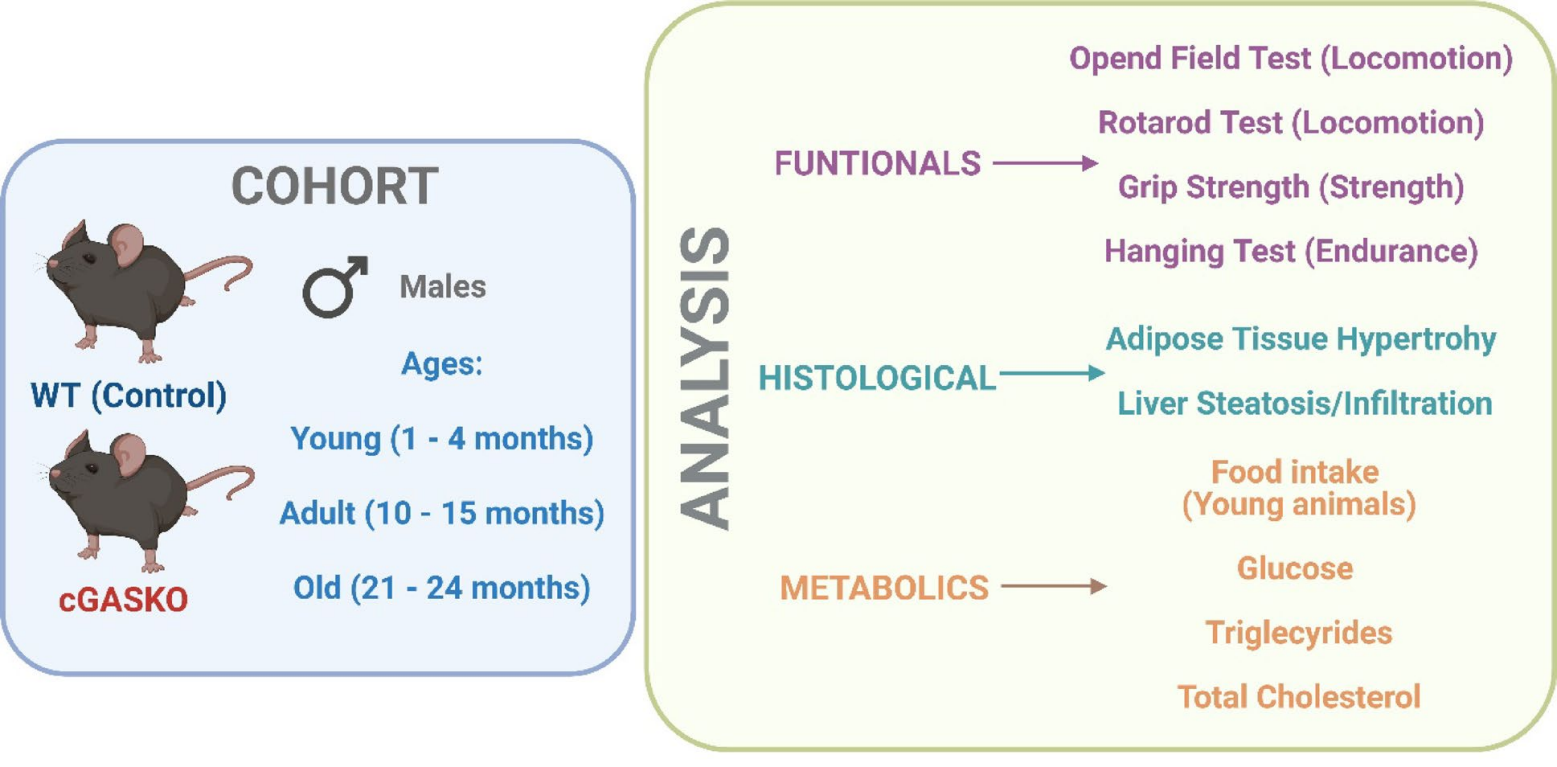
Diagram summarizing the experimental strategies employed in this investigation. The study cohort consists of male wild-type mice as controls and male cGASKO mice as the experimental group, which exhibit a complete absence of cGAS protein expression in all tissues. Both animal cohorts were employed at three developmental stages: young, adult, and old. The cohorts of mice underwent analyses classified into three categories: functional, histological, and metabolic. The functional analysis involves performing evaluations to measure the locomotor activity and strength of the mice. Histological analyses were conducted on gonadal adipose tissues and hepatic samples from the WT and cGASKO animals at three specified ages. In these tissues, adipocyte hypertrophy, lipid accumulation, hepatic steatosis, and cellular infiltration were evaluated. The metabolic assays included evaluating the intake of food in WT and cGASKO mice during young age, together measuring circulating levels of glucose, triglycerides, and total cholesterol across all ages. Created by Biorender.com

Body weight was measured using a weight scale on live male WT and cGASKO mice of predetermined ages, obtained from maintenance colonies, some of which were employed for further research in this and subsequent publications. A total of 20 WT mice and 20 cGASKO mice were employed throughout all evaluated ages. Over the course of a 15-week longitudinal investigation, young males at 3 months of age, representing both genotypes, were subjected to weekly weighing. A total of 15 WT mice and 16 conditional cGASKO mice were employed. The *n* values for endpoint evaluations in young, adult, and old ages mice are detailed in the legend of Fig. 1.

### Food consumption assessment

Food intake was assessed weekly by providing 200 g of food per four-animal cage and subsequently weighing the remaining food after a seven-day interval [22]. The overall consumption (delta) was divided by four to determine the consumption per animal [22]. This treatment was performed weekly for a period of 15 weeks to facilitate longitudinal assessment [22]. A total of 15 WT mice and 16 cGASKO mice were employed. This evaluation was conducted just within the young (1–4 months) cohort.

### Locomotor tests

**Fig. 2:**
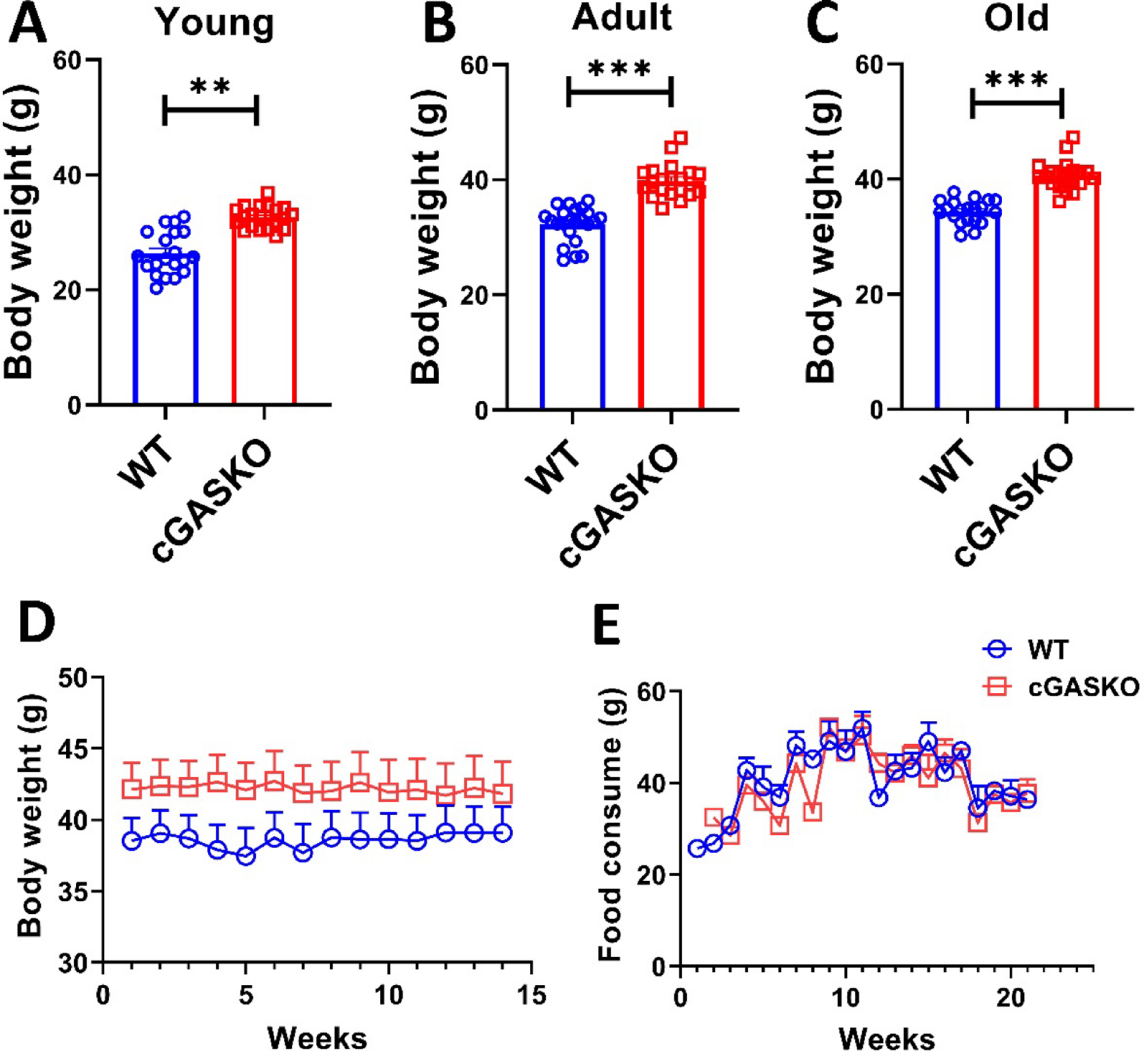
cGASKO mice demonstrate a persistent, lifelong elevation in body weight that cannot be attributed to higher intake of food during early life. Body weight (g) was documented in **A** young (1–4 months), **B** adult (10–15 months), and **C** old (21–24 months) male WT (blue) and cGASKO (red) mice. Each group comprised WT (*n* = 20) and cGASKO (*n* = 20) animals. Data are shown as mean ± SEM; significant differences were evaluated using an unpaired Student’s *t*-test (t_38_ = 3.12, **p = 0.003 and t_38_ = 4.55, ***p < 0.001). **D** Longitudinal body weight measurements were conducted on 15 WT mice and 16 cGASKO mice, starting at 3 months of age and evaluated weekly for a period of 15 weeks. Data were evaluated using one-way ANOVA (F_1, 30_ = 15.7, *p* < 0.001). **E** Average food consumption (g/day/mice) was assessed for the exact 15-week period (*n* = 15 WT, *n* = 16 cGASKO). Results are expressed as mean ± SEM; one-way ANOVA (F_1, 30_ = 0.89, *p* = 0.35)

**Fig. 3:**
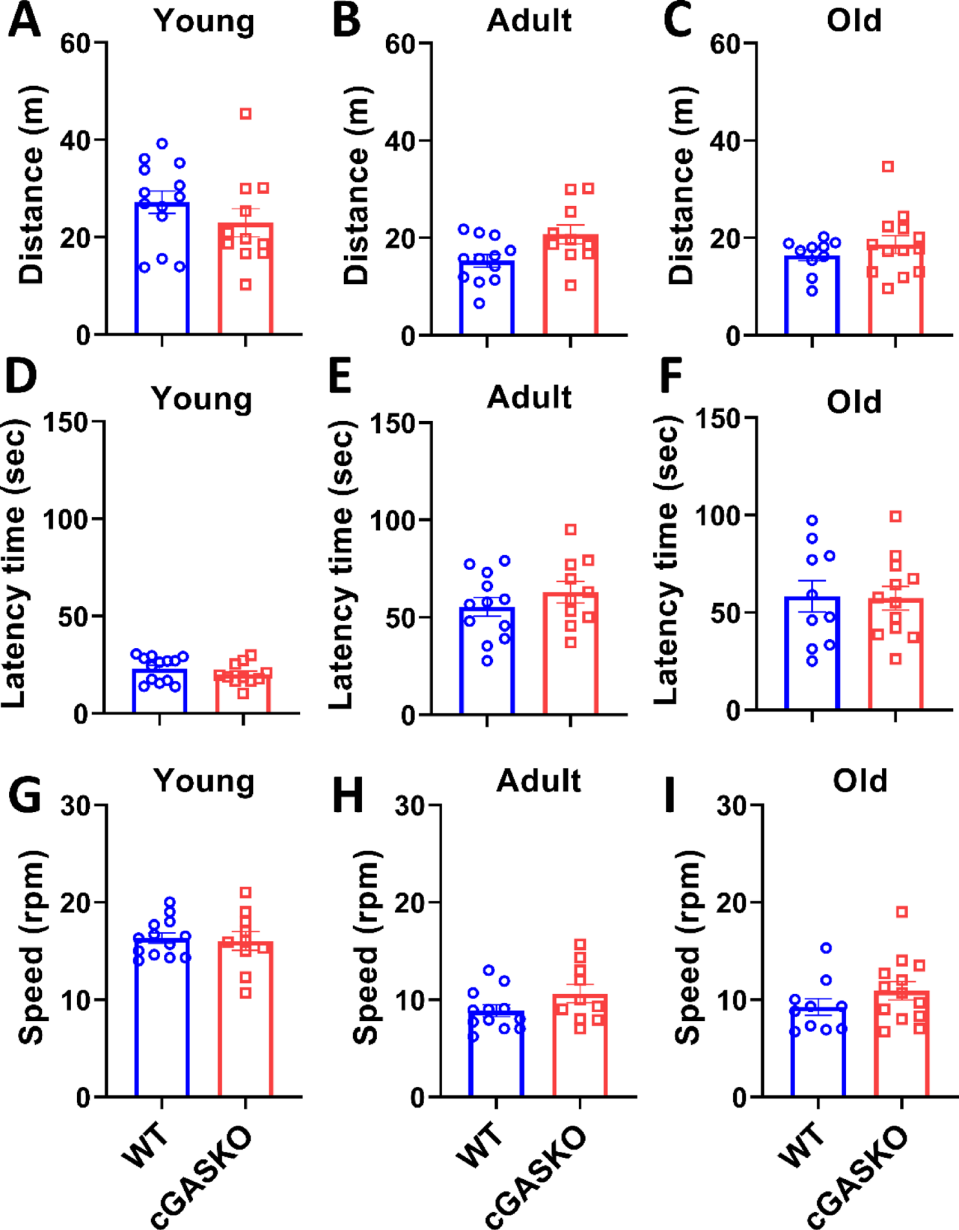
cGAS deficiency results in reduced relative muscle strength, while absolute strength remains unaffected. Grip strength (grams) was assessed in **A** young, **B** adult, and **C** old WT and cGASKO mice. WT (*n* = 7, 12 and 12, for each respective age); cGASKO (*n* = 9, 10 and for each respective age). Data are show as mean ± SEM; the Mann–Whitney U test s revealed no significant differences for young (U = 24.5, *p* = 0.4852) or old mice (U = 28.5, *p* = 0.0671) but was significant for adult mice (U = 28, **p = 0.0341). Strength-to-body weight ratio (g/g) in WT and cGASKO mice at **D** young, **E** adult, and **F** old ages. WT (n = 7, 12 and 12 respectively); cGASKO (n = 9, 10 and 16, respectively). Data are presented as mean ± SEM; the Mann–Whitney U test revealed significant differences for young (U = 1, ***p = 0.0003) and old mice (U = 25, *p = 0.0394), but not for adult mice (U = 16, *p* = 0.0668). Hanging test performance (latency to fall in seconds) in WT and cGASKO mice at **G** young **H** adult, and **I** old ages. WT (*n* = 8, 9 and 10, respectively); cGASKO (*n* = 10, 9 and 8, respectively). The Log-rank test was employed to compare survival curves. The analysis indicated no significant difference for young mice (X^2^ = 2.241, *p* = 0.1197) but revealed highly significant differences in adult (X^2^ = 69.57, ****p = 0.0001) and in old mice (X^2^ = 67.65, ****p = 0.0001)

In our study, the term “locomotor tests” refers to the behavioral evaluations utilized to evaluate motor activity, coordination, and strength in mice [27], including grip strength, hanging, open field, and rotarod tests. These are validated techniques for assessing motor functions without the inclusion of stress-induced metabolic variables [28, 29]. All strength evaluations were performed during the light phase under regulated temperature (22 ± 2 °C) and humidity conditions. To mitigate stress-related variability, animals were habituated to the apparatus 24 h prior to experimentation. Locomotor evaluations were conducted on three different age groups (young, adult, and old) in separate cross-sectional analyses, as specified by the *n* value in the legends of Figs. 2 and 3.

**Fig. 4:**
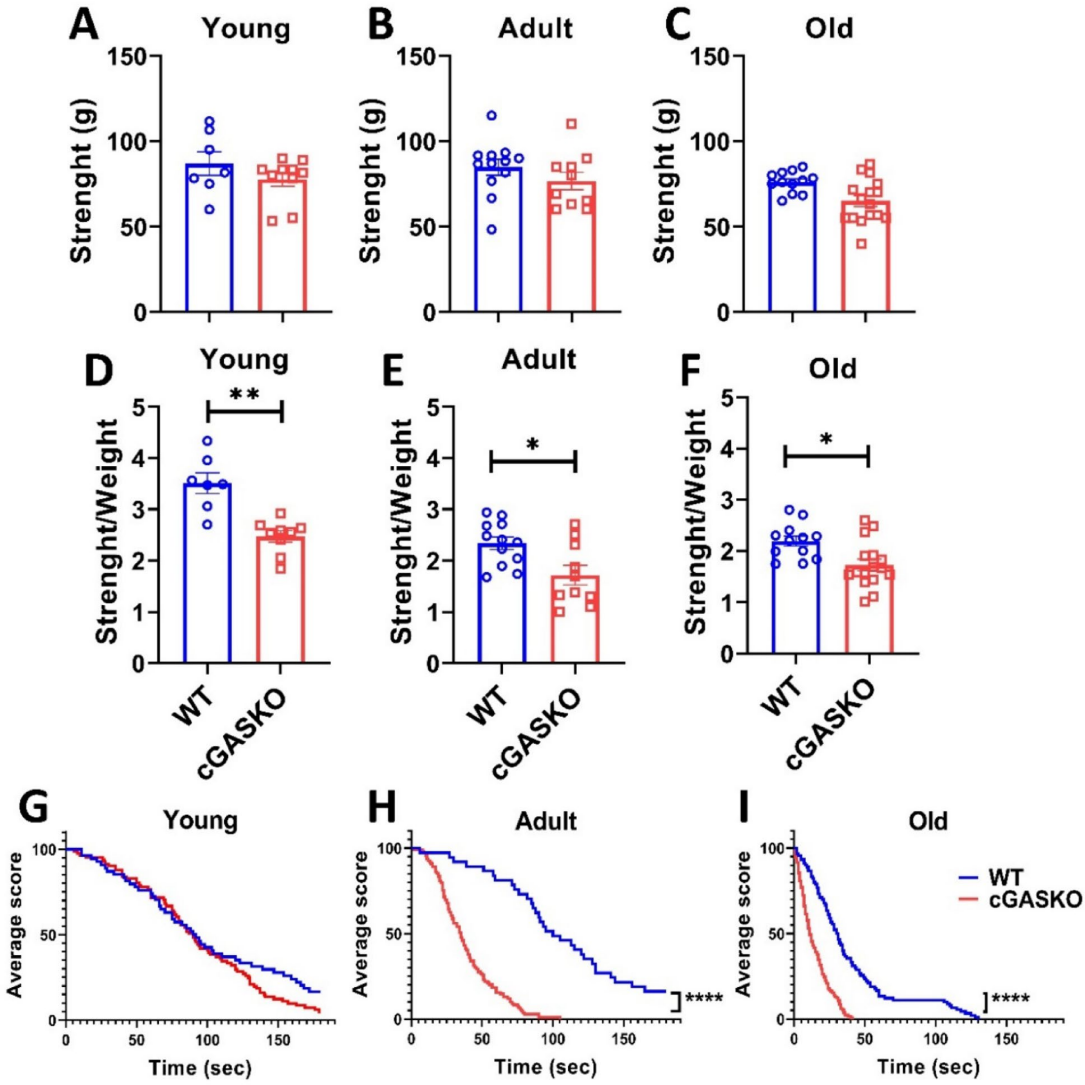
cGAS deficiency does not influence voluntary locomotor activity or exploration behavior across different ages. Spontaneous locomotor activity (Total distance traveled in cm) evaluated via the Open Field test assessed in **A** young (1–4 months), **B** adult (10–15 months), and **C** old (21–24 months) WT (blue) and cGASKO (red) male mice. (*n* = 13, 12, 10, in WT mice, and n = 11, 10 and 13 cGASKO mice (at each respective age). Latency to movement onset (s) was analyzed in **D** young, **E** adult, and **F** old mice. Average movement speed (cm/s) quantified in **G** young, **H** adult, and **I** old mice. Data are expressed as mean ± SEM. Mann–Whitney U test revealed no significant differences for any group or parameter: (A) U = 51, *p* = 0.2518; (B) U = 30, *p* = 0.0503; (C) U = 23, *p* = 0.1791; (D) U = 23, *p* = 0.9015; (E) U = 10.5, *p* = 0.2576; (F) U = 20, *p* = 0.3132; (G) U = 17.5, *p* = 0.6503; (H) U = 8.5, *p* = 0.1426; (I) U = 19.5, *p* = 0.2755

Forelimb grip strength. Grip strength of the forelimb. The forelimb grip strength of each experimental group was evaluated using the previously described method [22, 30]. Each animal was positioned on a grid connected to a dynamometer that measured the strength of its forelimbs. The procedure was performed three times for each animal, and the average force was estimated. We calculated the ratio of grip strength to body mass for each animal to evaluate the association between these two factors. The total number of young WT and young cGASKO mice was 7 and 12, respectively; the number of adult WT and cGASKO mice was 12 and 10, respectively; and the account of old WT and cGASKO mice was 12 and 16, respectively (Fig. 4).

*Hanging Test*: We additionally evaluated the general physical strength of each trial group using this method of testing [22, 31]. Mice were placed at the center of a grid and sustained their position with their forelimbs for 180 s. The number of falls, limited to 10, was registered. An analysis of the Kaplan–Meier curve was conducted to determine the duration until fall starts [22]. The sample size for young WT and cGASKO mice was 8 and 9, respectively. The total number of adult WT and cGASKO mice was 9 per group, and the sample totals for old WT and cGASKO mice were 10 and 8, respectively.

*Open field test (OFT)*: This test was carried out to measure anxiety-like behaviors and locomotor activity in animals [22, 32]. In summary, we placed the animals in a box of 40 cm in length, 40 cm in width, and 40 cm in height. We recorded and evaluated the total distance traveled (y-axis) in meters for each experimental animal group over a 10-min period using ANY-maze v6.34 software. A total of 13 young WT mice and 11 young cGASKO mice were utilized; 12 adult WT mice and 10 adult cGASKO mice were employed; and 13 old WT mice and 11 old cGASKO mice were included in the study.

*Rotarod test*: This evaluation assessed performance in response to forced exercise [22, 31]. Animals were set up on a rod that initially rotated at a constant speed of 4 rpm; once all the mice were positioned, the rod sped from 4 to 40 rpm over a period of 300 s. Subsequently, we quantified the latency time, defined as the period (in seconds) that the mouse maintains its balance on the rod during the test. The study involved 13 young WT mice, 10 young cGASKO mice, 12 adult WT mice, 10 adult cGASKO mice, 10 old WT mice, and 13 old cGASKO mice.

### Morphometric and histological analysis

Following to weighing the mice, they were anesthetized in a chamber with 3% isoflurane for tissue collection. Subsequently, they were exsanguinated via cardiac puncture utilizing 25 ml of saline solution per mouse [22]. The colon, gonadal adipose tissue, and liver were surgically removed [22]. A total of 10 WT mice and 10 cGASKO mice were employed for the assessment of all tissues and ages analyzed.

*Colon length*: The colonic segment from each animal within the indicated experimental groups was excised, and its length was subsequently measured using a ruler (centimeters) [22].

*Adipose and hepatic tissue*: The gonadal adipose tissue, described as a type of visceral fat in mice [33], was excised (except the testicles), weighed, and measured for length and width [22, 34]. The portion of this tissue that was farthest from the testis was excised for histological analysis [22]. Consequently, Eqs. 1 and 2 were utilized to obtain a measurement of abdominal adipose tissue area [22]. Initially, the weight (in grams) (W) was multiplied by the product of length and width (in centimeters) (A1) as per Eq. 1. An area of abdominal adipose tissue was determined by normalizing this new value (A1) relative to each animal’s weight (AW) in grams (Eq. (2)).

1 $$ {\text{P }}\left( {\mathrm{W}} \right){\text{x length }}\left( {{\mathrm{cm}}} \right){\text{x width }}\left( {{\mathrm{cm}}} \right) = {\mathrm{A}}1{ }\left( {{\mathrm{g}} \times {\mathrm{cm}}^{2} } \right) $$

2 $$ {\mathrm{Normalization}} = \frac{{{\mathrm{A}}1{ }\left( {{\mathrm{g}} \times {\mathrm{cm}}^{2} } \right)}}{{{\text{AW }}\left( {\mathrm{g}} \right)}} $$

**Fig. 5:**
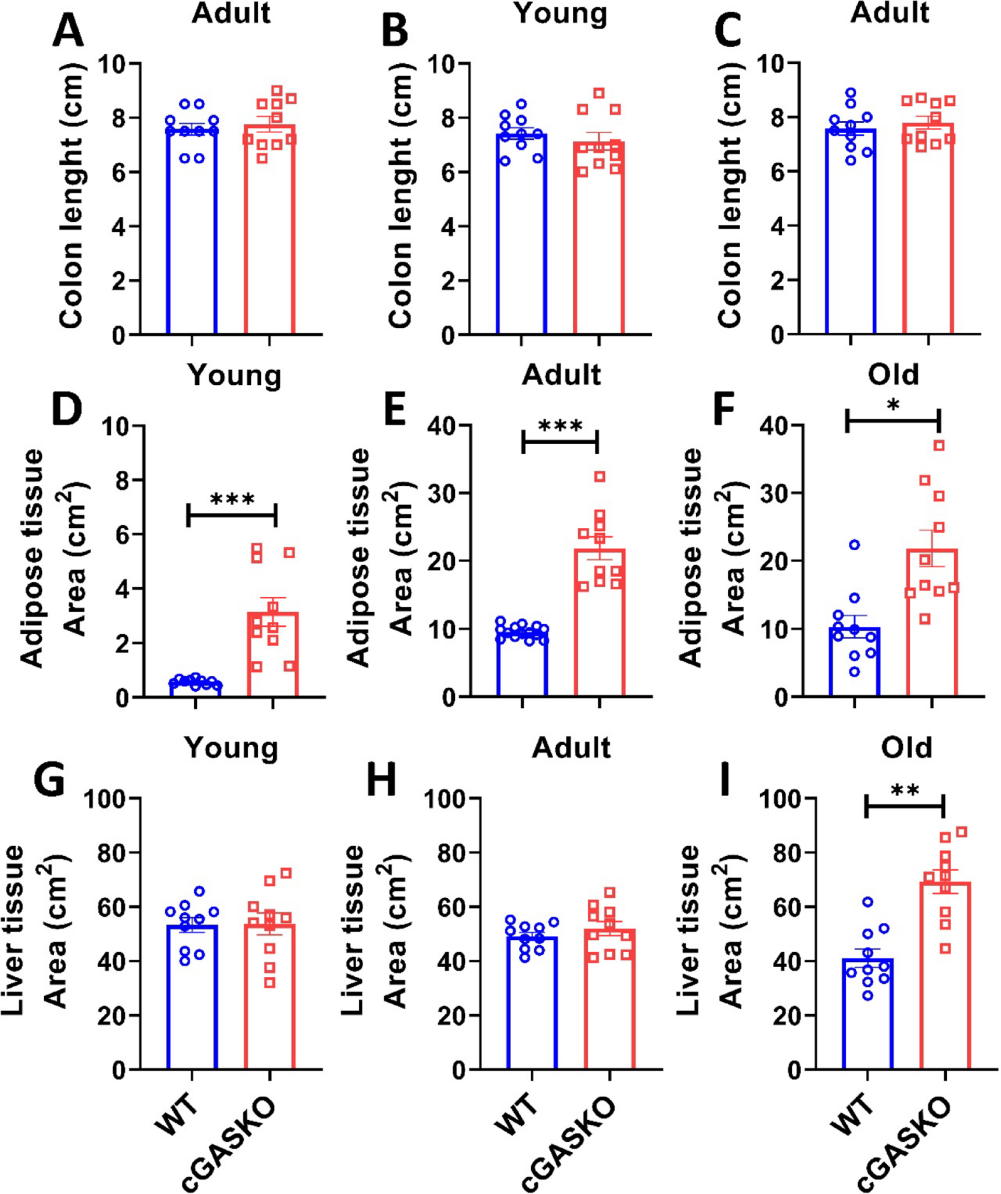
cGASKO mice exhibit no differences in colon size, hepatomegaly at old age, and an increased relative tissue area in adipose throughout studied ages. Colon length (cm) was measured in **A** young, **B** adult, and **C** old WT and cGASKO mice (*n* = 10 for each experimental group). Data are expressed as mean ± SEM; unpaired The Student’s *t*-test revealed no significant differences. Relative gonadal adipose tissue area index (A_1_/AW) in WT and cGASKO mice at **D** young, **E** adult, and **F** old ages (*n* = 10 per group). Data are expressed as mean ± SEM; unpaired Student’s *t*-test (t_18_ = 2.11, *p = 0.048; t_18_ = 2.89, **p = 0.009; t_18_ = 3.78, ***p = 0.001). Relative hepatic area index (A_1_/AW) in WT and cGASKO mice at **G** young, **H** adult, and **I** old ages (n = 10 per group). Data are expressed as mean ± SEM; unpaired Student’s *t*-test (t_18_ = 2.83, **p = 0.011; t_18_ = 2.89, **p = 0.009; t_18_ = 3.57, **p = 0.002)

The distal end (0.5 × 0.5 cm) of the excised gonadal adipose tissue, situated farthest from the testes, was preserved in 4% paraformaldehyde (4% PFA) (Sigma) for 24 h at 4 °C. Subsequently, it was embedded in Tissue-Tek O.C.T. compound for cryosectioning and subsequently stored at − 80 °C until it was required for use [22]. The LEICA CM1950 cryostat was utilized to obtain histological sections with a thickness of 12 μm. The sections were then stained with a Hematoxylin & Eosin [H&E] solution (Sigma). Five sections from each adipose tissue sample of each animal were collected for obtaining 10 pictures per field of view at 40 × magnification using a Nikon ECLIPSE E200LED MV R bright-field microscope (Fig. 5A). The average adipocyte area was calculated by measuring N ≥ 400 cells from five random fields of each picture (10 images per mouse at 10 × magnification) (Fig. 5B). The adipocyte area was analyzed using ImageJ software.

The caudate lobe was excised from each animal following the measurement of liver weight, length, and width [22, 34]. The lobe was dissected and fixed in 4% PFA (Sigma) at 4 °C for 24 h [22]. Subsequently, histological processing was conducted utilizing the Thermo Microm STP120 tissue processor, followed by embedding in a Thermo HistoStar paraffin block. The Leica RM 2125 RTS microtome was utilized to obtain slices measuring 3 µm in thickness. Thereafter, the slices were stained using H&E (Sigma) solution. A total of 5 WT and 5 cGASKO mice were utilized to assess this tissue across all ages.

### Biochemical parameters

The animals in each experimental group, following a 4-h fast [22, 35], were restrained for blood collection from the tail using a scalpel. 50 µm of this whole blood sample were utilized for glucose measurement using an Abbott Freestyle Optium glucose meter with Abbott Freestyle Optium test strips (mg/dl). The leftover volume of the whole blood sample (200 to 300 µl per animal) was centrifuged at 2500 rpm for 15 min at ambient temperature using an Eppendorf 5415C centrifuge Thermo Scientific™ Fresco™ 17, and the supernatant (serum) was collected. Total cholesterol and triglycerides were measured from this supernatant using 100 μl of blood with LiquiColor® commercial kits, both obtained from the same supplier. Assay functionality was confirmed through the application of a multi-point calibration standard curve, comprising blank samples (prepared by mixing solutions devoid of standard or serum sample) and serially diluted standards of known concentration supplied by the manufacturer, serving as the technical positive control to validate the linearity and precision of measurements for each metabolite analyzed. Absorbances were measured using a Tecan Infinite® 200 PRO NanoQuant spectrophotometer at a wavelength of 546 nm. This work employed a total of 6 WT mice and 6 cGASKO mice across all age categories, with the experimental sample size determined by the availability of kits. The subsequent table presents the means, standard deviations (SD), and coefficients of variation (CV) for each biochemical parameter (Table 1).

**Table 1:** Mean, Standard Deviation, and Coefficient of Variation of Serum Glucose, Triglycerides, and Total Cholesterol from WT and cGASKO mice at different ages

		Glucose			Triglycerides			Total Cholesterol		
Ages	Group	Mean (mg/dL)	SD (mg/dL)	CV (%)	Mean (mg/dL)	SD (mg/dL)	CV (%)	Mean (mg/dL)	SD (mg/dL)	CV (%)
Young	WT	175.8	17.7	10.0	166.4	13.6	8.1	64.0	2.4	3.7
	cGASKO	149.5	13.0	8.7	191.2	8.6	4.5	121.0	21.9	18.1
Adult	WT	149.0	19.7	13.2	166.0	16.5	10.0	69.4	14.2	20.5
	cGASKO	150.4	13.8	9.2	162.8	4.7	2.9	150.5	15	10.0
Old	WT	134.8	10.3	7.6	162.4	3.6	2.23	65.3	10.4	15.9
	cGASKO	137.8	17.3	12.6	151.4	13.16	8.7	130.8	26.5	20.2

### Data analysis

The particular *n* values for each experimental group are provided in the corresponding Figure Legends and previously in the Methods section. The experimental sample size, represented as *n*, was established based on the availability of animals of varying ages, the time necessary for conducting each experiment, and the accessibility of reagents for measurements, aiming to minimize animal usage in alignment with ethical principles. Data are expressed as mean ± standard error of the mean (SEM). Statistical analyses were performed using GraphPad PRISM v8.0 (GraphPad Software, San Diego, CA, USA). The Shapiro–Wilk test was utilized to evaluate the normality of the data. Levene’s test was utilized to assess the homogeneity of variances. The choice between parametric and non-parametric statistical methods was determined by the outcomes of this test. Given the restricted sample sizes in specific longitudinal cohorts, robust non-parametric tests were utilized when the assumptions of parametric tests could not be definitively substantiated. The unpaired Student’s *t*-test was utilized for two independent groups when normality was met; otherwise, the non-parametric Mann–Whitney U test was performed. A one-way Analysis of Variance (ANOVA) was employed for three or more independent groups with normally distributed data, whereas the non-parametric Kruskal–Wallis test was utilized for non-normally distributed data. The survival study of time-to-event data (Hanging Test) employed Kaplan–Meier survival curves, with significance evaluated using the Log-rank test. Statistical significance was determined at *p ≤ 0.05, **p ≤ 0.01, ***p ≤ 0.001, and ****p ≤ 0.0001.

### Flowchart of methodology

Figure 1 delineates the structural framework of the study. Cohorts of WT and cGASKO male mice (young, adult, and old) were formed for longitudinal observation of body weight. The research methodology included metabolic evaluations (dietary intake during early development, serum cholesterol, triglycerides, and glucose levels), histological analysis of adipose and hepatic tissues (hypertrophy and lipid deposition), and functional assessments of gross locomotion (Open Field, Rotarod) and muscular strength/endurance (Grip Strength, Hanging Test) at specified age milestones.

## Results

### Lifelong body weight increase in cGASKO mice: not attributed to early-life food intake

While it has been demonstrated that the absence of STING expression inhibits changes in lipid metabolism and body weight in animal models of obesity [13, 36, 37] it remains unassessed whether cGAS influences body weight under physiological conditions during life. To assess potential differences in body weight between WT (control) and cGASKO (experimental group) mice throughout their lifespans, weight measurements were obtained at various age categories: young (1 to 4 months), adult (10 to 15 months), and old (21 to 24 months). WT mice exhibited an average weight of 26.7 ± 3.76 g (*n* = 20) at a young age, 31.6 ± 0.68 g (*n* = 20) as adults, and 35.6 ± 1.78 g in old age (n = 20). In contrast, cGASKO mice displayed weights of 33.9 ± 0.99 g (*n* = 20), 40.7 ± 1.12 g (*n* = 20), and 40.0 ± 0.86 g (*n* = 20) in the respective age groups (Fig. 2 A-C). The results demonstrate that cGASKO mice exhibited significantly increased body weight than WT mice across all assessed age groups (Fig. 2 A-C). Subsequently, we examined food consumption to ascertain the fundamental cause of the weight differential between WT and cGASKO mice across time during young age. Young mice, aged 3 months, were monitored for weekly weight and food consumption throughout a period of roughly 4 months, culminating in an age of 7 months at the study’s conclusion. An insignificant average weight disparity of approximately 5 g was noted between WT and cGASKO mice in the body weight assessment (Fig. 1D). Furthermore, the examination of food intake indicated no substantial differences between the two groups (Fig. 2E). The observations indicate that the increased body weight in cGASKO mice is not influenced by food intake in young mice; nevertheless, the role of food consumption in later life stages remains unclear. The weight disparity between WT and cGASKO mice remains consistent across all examined ages.

Furthermore, as cGASKO mice continuously demonstrate elevated body weight at all ages relative to WT (control) mice, we examined whether this disparity may be attributed to augmented muscle mass and, subsequently, enhanced strength. Forelimb grip strength was measured with a dynamometer to evaluate potential variations in force. The mean peak force delivered by each animal during three trials was documented and represented (Fig. 3A-C). No notable variations in grip strength were detected between WT and cGASKO mice, nor were there any age-related effects (Fig. 3A-C). Moreover, when grip strength is normalized to body weight, statistically significant differences were seen between cGASKO mice and WT mice across all examined ages (Fig. 3D-F). The findings indicate that cGASKO mice do not display a significant increase in absolute muscle strength or mass. Nevertheless, when assessing strength normalized to body weight, these animals exhibit a reduced delta, suggesting that the foreleg strength of cGASKO mice decreases relative to body mass compared to WT mice across all assessed ages.

To assess variations in the ability to support their own body weight among the experimental groups, we conducted the hanging test. The incidence of falls for each mouse was documented, and the mean values for each experimental group were illustrated (Fig. 3G-I). The incidence of falls increased with age across all experimental cohorts. At every evaluated age, cGASKO mice demonstrated an elevated number of falls than WT (control) mice (Fig. 3G-J). The results indicate that cGASKO mice possess a diminished capacity to sustain their elevated body mass over prolonged periods. The compromised weight-bearing ability may be attributed to the possible biomechanical impact of their increased body weight compared to weight control group.

Additionally, to evaluate possible locomotor deficits, mice performed the Open Field Test (OFT). No statistically significant differences were observed between the groups or among the different age groups (Fig. 4A-C). Consequently, our data suggests that general locomotor activity remains stable, which would influence their capacity for moving around the OFT arena during the analyzed timeframe. To validate this finding, we subsequently conducted the Rotarod test to evaluate locomotor ability and possible sedentary behavior. Our results revealed no statistically significant changes in latency time among experimental groups across all evaluated age groups (Fig. 4D-F). Similarly, the analysis of running speed revealed no variation between cGASKO and WT mice (Fig. 4G-I). Correlating this result with the findings from the hanging test suggests that, although general motor coordination remains intact, cGASKO mice likely have higher difficulties supporting their body weight compared to WT mice across all studied ages (Fig. 3D). These data collectively suggest that the motor deficit is specific to weight-bearing performance and does not reflect an increase in sedentarism or generalized locomotor dysfunction in cGAS-deficient animals.

### cGAS mice exhibit increased accumulation of lipids in adipocytes over all age groups

Animals exposed to a high-fat diet (HFD) exhibit alterations in the gastrointestinal system, characterized by an unchanged length of the small intestine (the site of fat absorption) and a decrease in colon length [38]. Consequently, we evaluated the colon length in cGASKO mice to determine any modifications. No significant differences were seen among the experimental groups (Fig. 5A-C), suggesting that cGAS-deficient mice display no diet-related, inflammatory, or gastrointestinal alterations [38].

In addition to a difference in body weight, macroscopic inspection of cGASKO mice demonstrated a notable increase in adipose tissue (fat pad mass) relative to WT control animals throughout all age groups (data not shown). This qualitative observation, evident upon dissection, supports the notion of extensive lipid accumulation, which was further investigated at the cellular level. We measured the gonadal adipose tissue of each experimental group and observed that cGASKO mice displayed a significant enlargement in adipose tissue area compared to WT mice at all age points (Fig. 5D-F). Furthermore, we assessed the hepatic region in both experimental cohorts to ascertain the presence of hepatic steatosis. The liver size in cGASKO animals remained comparable to that of WT counterparts during both young and adult ages (Fig. 5G-H). In old cGASKO mice, an increase in liver size was observed compared to old WT mice (Fig. 5I). These findings suggest that the increased body weight observed in cGASKO mice may result from enhanced lipid deposition within adipose tissue throughout their lifespan.

**Fig. 6:**
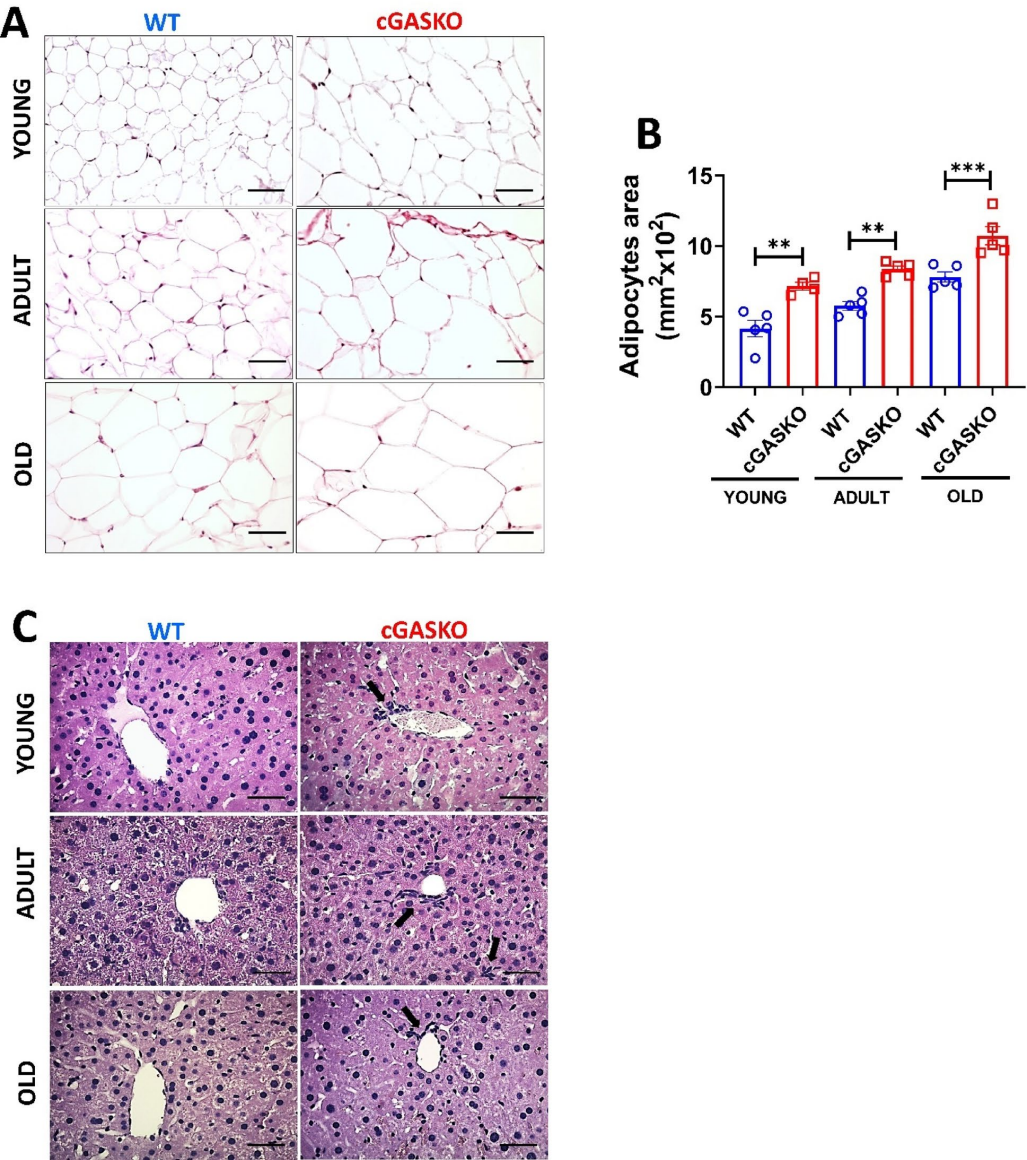
cGASKO mice demonstrate adipocyte hypertrophy and hepatic cellular infiltration across all ages. Representative images from H&E staining of **A** gonadal adipose tissue and **C** liver sections from WT (blue) and cGASKO (red) mice at different ages (young, adult, old). Quantification of adipocyte area (µm^2^) **B** utilizing ImageJ. The average adipocyte area was assessed by measuring N ≥ 400 cells from 5 random fields of view per mouse: WT (n = 5, 5 and 5, for each age); cGASKO (*n* = 4, 5 and 5, for each age). Data are shown as mean ± SEM; one-way ANOVA indicating significant differences (F_1, 26_ = 10.2, **p = 0.004; F_1, 26_ = 12.9, ***p < 0.001; F_1, 26_ = 15.4, ***p < 0.001). Representative images in (**C**) illustrate hepatocyte morphology and cellular infiltration (shown by black arrows). The bar represents 100 µm in all imagines

To confirm that the increased adipose tissue area in cGASKO mice was due to enhanced lipid accumulation, we analyzed the fat content in the parenchymal cells of this tissue and the liver. We employed histological sections to evaluate the adipocyte tissue (Fig. 6A) and the area of adipocytes (Fig. 6B). Compared to WT mice, cGASKO mice exhibited a significant enlargement of adipocyte area, particularly within the old age group (Fig. 6A, B). Nonetheless, cGASKO mice do not demonstrate variations in hepatic accumulation of lipids when compared to WT mice. However, in cGASKO mice throughout all assessed ages, there is a smallest increment in cellular infiltration (Fig. 6C). The findings suggest increased lipid accumulation within the adipose tissue of cGASKO mice compared to WT mice.

### cGASKO mice display elevated circulating total cholesterol levels throughout life

**Fig. 7:**
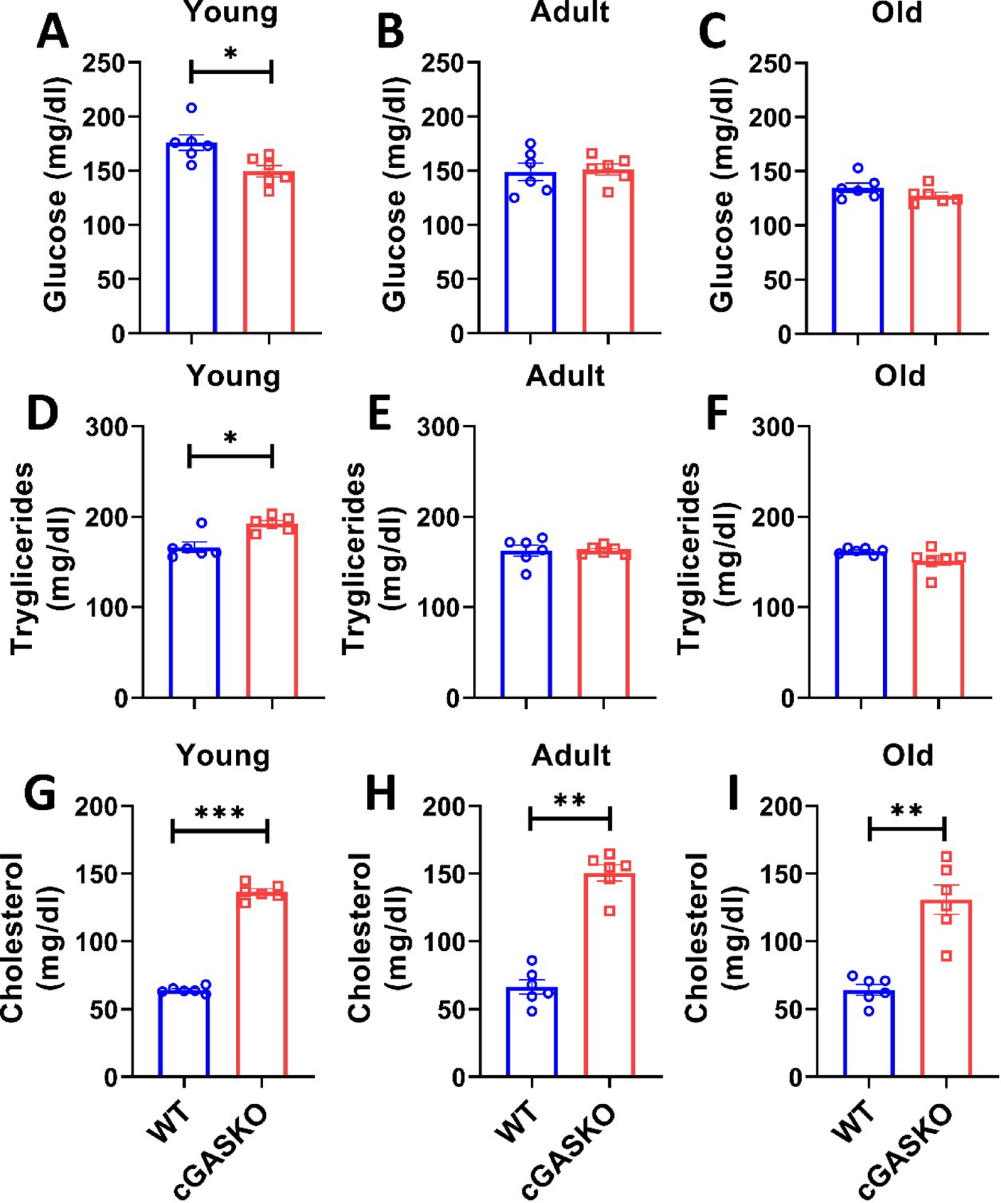
cGASKO mice display transient fluctuations in glucose and triglycerides levels alongside a substantial increase in total cholesterol across different ages. Blood glucose levels (mg/dl) in **A** young, **B** adult, and **C** old WT and cGASKO mice (n = 6 per experimental group). Data are represented as mean SEM; unpaired Student’s *t*-test (t10 = 2.39, *p = 0.038). Triglyceride levels (mg/dl) in WT and cGASKO mice at **D** young, **E** adult, and **F** old ages (n = 6 per group). The data are mean SEM; unpaired Student’s *t*-test (t_10_ = 2.34, *p = 0.04). Total cholesterol (mg/dl) measured in young, **H** adult, and **I** old WT and cGASKO mice (*n* = 6 per experimental group). Data are expressed as mean SEM; one-way ANOVA (F_1, 10_ = 8.5, **p = 0.015; F_1, 10_ = 10.2, **p = 0.009; F_1, 10_ = 11.7, ***p = 0.006)

In order to determine if the increased fat accumulation in adipose tissue is associated with enhanced circulating lipids in cGASKO mice, we evaluated glucose, triglycerides, and total cholesterol (Fig. 7). The findings revealed a difference in circulating glucose levels between WT (175.83 ± 3.22 mg/dl) and cGASKO (149.50 ± 2.45 mg/dl) mice at a young age (Fig. 7A). No significant variations in glucose levels were noted between WT and cGASKO animals at adult (149.00 ± 3.21 and 150.40 ± 2.67 mg/dl, respectively) and old age (134.83 ± 2.12 and 130.80 ± 2.44 mg/dl, respectively) (Fig. 7B-C). Upon assessment of triglyceride levels (Fig. 7D-F), WT mice showed values of 166.40 ± 2.54, 165.97 ± 2.91, and 163.46 ± 1.62 mg/dl at young, adult, and old age groups, respectively. In contrast, cGASKO mice exhibited mean triglyceride levels of 191.22 ± 1.55, 162.82 ± 1.23, and 160.10 ± 2.53 mg/dl across the same age groups (Fig. 7D-F). Significant variations were observed in cholesterol levels (Fig. 7G-I). Total cholesterol levels in WT mice were around 64.02 ± 0.25, 66.37 ± 2.91, and 65.11 ± 2.84 mg/dl for the young adult, and old age groups, respectively (Fig. 7D-F). In contrast, cGASKO mice had total cholesterol levels of around 140.10 ± 1.89, 148.4 ± 3.93, and 130.81 ± 4.78 mg/dl at the corresponding ages (Fig. 7G-I). The research suggests that the absence of cGAS leads to temporary fluctuations in glucose and triglyceride levels during young age, which then normalize with time. The total cholesterol levels in cGASKO animals remain elevated throughout their lives compared to WT, suggesting that cGAS may substantially affect cholesterol homeostasis.

### Flowchart of results outcomes

**Fig. 8:**
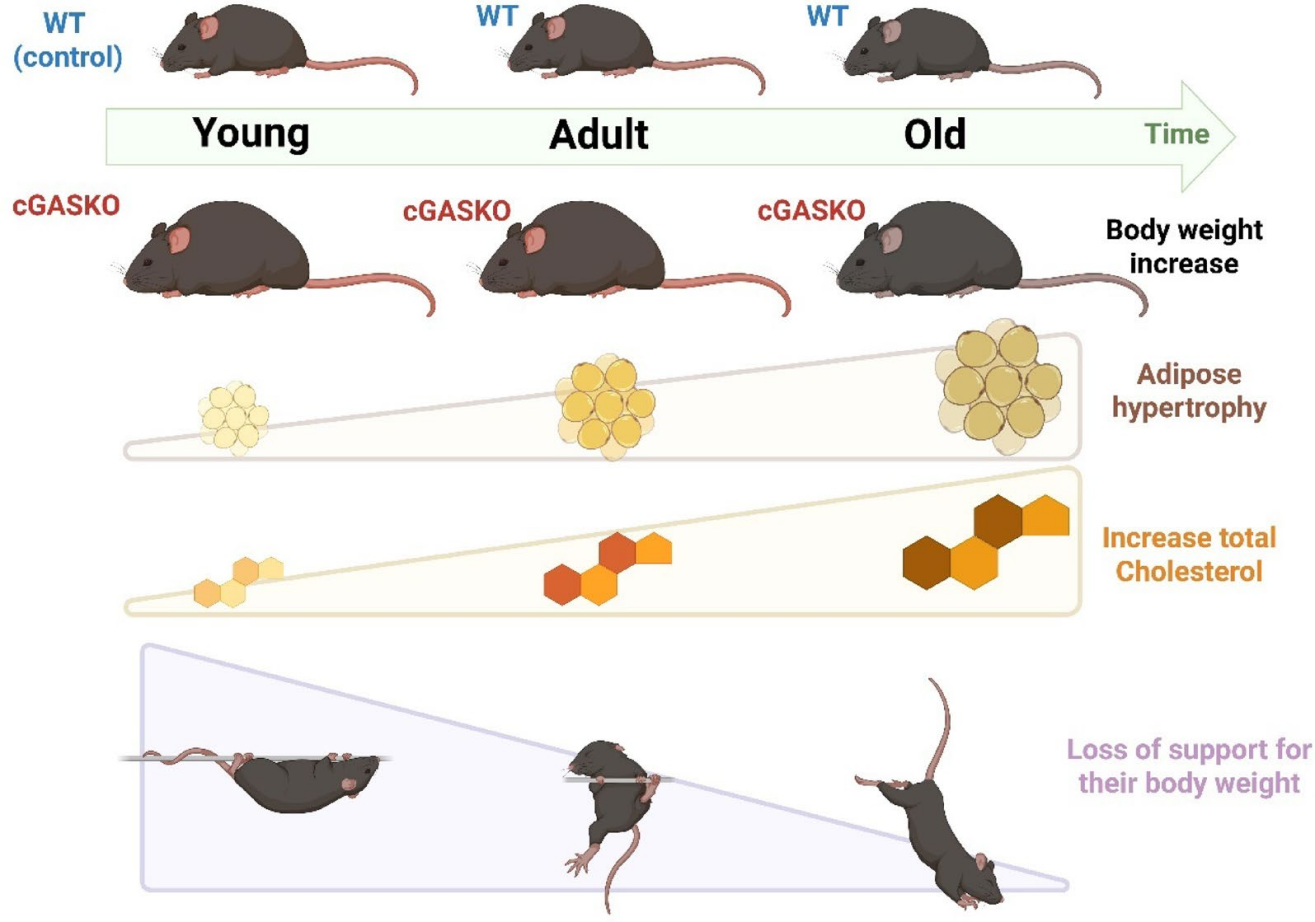
The diagram presents the principal findings of this research. The figure shows the most significant findings of the study**.** The ages of the WT and cGASKO male cohorts at the young, adult, and old ages are delineated. Throughout these periods, cGASKO mice (shown by the time-marking arrow) demonstrate an elevation in body weight, a larger amount of adipose tissue, and an accumulation of enhanced lipid levels, including an increase in circulating total cholesterol over time. cGASKO mice, contrast WT controls, fail to maintain their body weight over time, leading to a higher incidence of falls during the hanging test attributable to weight gain caused by the accumulation of lipids in adipose tissue. Created by BioRender.com

Figure 8 consolidates the principal data, illustrating the suggested correlations between cGAS deficiency and the reported observations. The lack of cGAS results in a persistent lifelong rise in body weight, mechanistically supported by adipocyte hypertrophy and lipid accumulation, resulting in higher circulating total cholesterol levels. The enhanced body mass correlates with diminished weight-bearing capacity (impaired strength and endurance), although general locomotor function remains intact, underscoring a distinct deficiency in muscle performance related to the increased load.

## Discussion

This study evaluated the effects of cGAS and cGAMP absence in mice on lipid metabolism throughout different ages while on a standard chow diet. The current literature mostly highlights the function of STING in this context [8–13]. Nonetheless, the roles of cGAS and/or cGAMP in these processes remain poorly investigated. Our data indicates that the absence of cGAS leads to an increase in body weight, despite unchanged food intake during young age and persistent general locomotor activity. This phenomenon is primarily attributed to an increase in adipose tissue mass and hypertrophy of adipocytes observed throughout the course of life of these animals. This finding highlights that cGAS dysfunction alone, regardless of the introduction of metabolic diseases or inflammatory stimuli, is sufficient to progressively compromise cholesterol and adipose homeostasis.

In contrast to our findings, prior research indicates that in mouse models of obesity and metabolic disorders, the administration of a STING agonist, analogous to cGAMP, which is a product of cGAS, diminishes inflammation, cellular damage, and lipid concentrations [39]. In contrast, some studies indicated that the absence of cGAS and cGAMP in obesity models results in decreased adipose fat accumulation and inflammation [9]. This underscores a vital distinction: the function of cGAS-cGAMP molecules in lipid metabolism may be contingent upon the organism’s metabolic environment [10]. Consequently, our research reveals a physiological, context-dependent function of cGAS that may be hidden in obesity-driven animals characterized by inflammatory signals.

Considering the observation that cGASKO mice demonstrated increased body weight compared to WT mice, we performed locomotor tests in order to determine whether the movement ability of these animals was impaired, potentially contributing to their weight gain. The locomotor assessments employed to evaluate strength and coordination in mice [27] include the open field test (spontaneous activity) [28], the rotarod test (balance and coordination under forced exercise) [29], the hanging test (muscular strength for body support) [29], and the grip strength test (forelimb strength and body support) [30]. Performance in the open field and rotarod assessments were similar between cGASKO and WT (control) mice, demonstrating maintained overall neuromuscular coordination and balance (general locomotion). Nonetheless, this consistency starkly contrasts with the results from the weight-bearing experiments; in the hanging and normalized grip strength evaluations, cGASKO mice exhibited a marked decline in their capacity to support their own body weight. This discovery strongly suggests a fundamental biomechanical restriction resulting from elevated adiposity and body mass in cGASKO mice. This conclusion is corroborated by the reduced expression levels of cGAS in neurons and Schwann cells under homeostatic conditions, indicating that a significant essential malfunction in the neuromuscular circuitry is unlikely [36, 40]. Therefore, while the core neuromuscular function remains intact, the animals’ effective strength is compromised due to increased body weight, indicating a functional defect. To definitively exclude the possibility of an inherent muscular deficit, additional research is required to isolate muscle performance from total body weight load.

Weight increase or obesity is generally associated with changes in the function of metabolic tissues, particularly the liver [3]. The livers of cGASKO mice have no evidence of steatosis; however, they do show cellular infiltration, as reported previously [20] (in press). Hepatomegaly was seen in old cGASKO mice, indicating potential organ failure relative to WT mice. Independent investigations indicate that cGAS depletion can intensify hepatic inflammation and cellular infiltration via many non-canonical mechanisms. In models of diet-induced Non-Alcoholic Steatosis Hepatic (NASH), cGASKO animals exhibit exacerbated steatosis, endotoxemia, and elevated monocyte/macrophage infiltration in the liver [41, 42]. Numerous studies indicate that cytosolic DNA detection via the cGAS-cGAMP-STING pathway not only stimulates type I interferons but also NF-kB-dependent chemokines [43]. Endogenous DNA sources can also activate additional sensors, including TLR9 and AIM2, which are alternate receptors to cGAS that enhance chemokine production and tissue invasion [41, 44]. Without precise characterization of cellular infiltration or identification of molecules, the physiological significance remains unclear, representing an important limitation of the work. Our data and literature findings offer a plausible rationale for the elevated hepatic infiltration noted in our cGASKO mice: (i) the absence of cGAS disrupts cellular homeostasis and DNA containment throughout life (see more details below), and (ii) it facilitates the activation of receptors that detect danger-associated molecular patterns (DAMP) resulting in inflammation [20, 41, 44].

On the other hand, in young cGASKO mice, a temporary or transient decline in glucose levels was observed at young age, followed by normalization, which may be directly associated with a non-canonical nuclear function of cGAS, independent of cGAMP synthesis [45]. Beyond its established cytosolic function, cGAS is recruited to the nucleus following phosphorylation, where it has been demonstrated to modulate the expression of proliferative genes in pancreatic β-cells by inhibiting the transcription factor CEBP-β [18]. The lack of cGAS may alleviate this inhibition, facilitating β-cell proliferation and insulin secretion, consequently decreasing blood glucose levels [18]. The normalization of glucose levels with age suggests that compensatory systems have been compromised by age-related metabolic stress, requesting future validation.

Analogous to glucose levels, the elevated circulating triglycerides observed in young cGASKO mice, which subsequently normalized with advancing age, constitutes a noteworthy age-dependent metabolic adaptation. This observation is in direct opposition with the chronic hypertriglyceridemia and hepatic steatosis observed in STINGKO mice [22], emphasizing that the total absence of cGAMP—rather than STING—induces unique regulatory responses. The initial increase has been suggested to indicate an early imbalance in hepatic lipid export, possibly resulting from disrupted AMPK–SREBP1c signaling, a pathway associated with cGAMP–STING activity [41]. Eventually, this initial dysfunction may be mitigated, since the depletion of cGAMP and the resulting less inflammatory signaling could facilitate a significant adaptive expansion of adipose tissue [46]. This mechanism parallels the juvenile immune tolerance observed in beige adipose tissue, wherein diminished IFN-β (a type I Interferon) signaling—potentially through IRF7 suppression or mtRNA activation of RIG-I/MDA5 sensors—promotes lipid storage and adipogenesis during early developmental phases [47]. The resultant adipocyte enlargement serves as an essential buffering reservoir that actively sequesters triglycerides, offering a compensatory mechanism for their clearance from circulation and averting hepatic steatosis [48, 49]. Subsequent research should examine these processes or others in cGASKO mice. The present study indicates a biphasic adaptation in which an initial imbalance is rectified by subsequent compensatory restructuring of peripheral lipid metabolism.

The persistent hypercholesterolemia in cGASKO mice constitutes a key finding of our research. This phenotype probably arises from the disruption of intricate feedback networks associated with cholesterol sensing. Recent findings indicate that the STING transmembrane domain contains two conserved cholesterol-binding motifs [11]. Normal cholesterol efflux or reduction from those sites facilitates the complex cGAMP-STING translocation from the ER to the Golgi for signaling activation. In the presence of elevated cholesterol levels, the complex cGAMP-STING is sequestered within the ER [11, 50]. In cGASKO mice, the continuous absence of cGAMP would inhibit the dynamic activation of STING, resulting in its sustained retention in the ER and disrupting ER-Golgi lipid exchange. This retention may result in anomalous accumulation of cholesterol and disruption of lipoprotein vesicular transport [51–53]. Simultaneously, an elevation in cellular cholesterol or oxysterol levels activates LXR, which stimulates the transcription of SMPDL3A, leading to the degradation of cGAMP [15]. In cGASKO mice, the complete lack of cGAMP may inhibit SMPDL3A from accessing its substrate, resulting in persistent LXR activation to compensate for the deficiency in degradation signals, hence enhancing cholesterol production. Furthermore, recent research by Deng et al. (2024) demonstrated that cGAMP interacts with Rab18, a small GTPase, which facilitates the mobilization of cholesterol from the endoplasmic reticulum [52, 53]. Without cGAMP, Rab18 may stay inactive, limiting lipid transport from the ER to lipid droplets and resulting in cholesterol accumulation. All these hypotheses need further assessment. Collectively, the evidence and suggested mechanisms collectively indicate that cGAMP probably regulates cholesterol levels.

The cGAS–cGAMP axis seems to exert indirect control over the enzymatic and transcriptional networks controlling lipid metabolism. To the best of our knowledge, no research has directly shown changes in the catalytic activity of enzymes in cGASKO mice. Evidence indicates that cGAS affects these pathways via mitochondrial integrity, NAD⁺ redox equilibrium, and nuclear signaling [41, 49, 54]. The lack of cGAS alters mitochondrial dynamics, resulting in ineffective β-oxidation of fatty acids [54]. This deficiency modifies the cellular redox state, diminishing the availability of NAD⁺ necessary for oxidative metabolism and transcriptional control [55]. Reduced NAD⁺ levels are recognized to block PGC-1α/PPARα signaling, a crucial regulator of fatty acid oxidation, and facilitate lipid accumulation in both hepatic and adipose tissues [55–57]. Moreover, cGAS deficiency results in the basal activation of AMPK and mTORC2, both of which are implicated in lipid synthesis and mitochondrial respiration [47, 54]. We propose that the persistent dyslipidemia and adipocyte hypertrophy are likely consequences of mitochondrial and transcriptional metabolic remodeling, rather than mere changes in enzyme levels or activity, induced by a permanent absence of cGAS-mediated genomic surveillance and redox homeostasis [14, 23, 49, 58].

The modifications to metabolism observed in our research cannot be comprehensively understood without recognizing the essential role of cGAS in maintaining genomic integrity and its association with aging [45, 59–62]. In addition to its cytoplasmic function in detecting dsDNA, cGAS is also found within the nucleus, where it contributes to chromatin organization and DNA repair processes [58, 63, 64]. These interactions inhibit improper transcription and preserve heterochromatin stability [20] (in press). The absence of cGAS affects homeostatic regulation, resulting in an accumulation of dsDNA and persistent activation of alternative innate immune sensors, which maintain a chronic low-grade pro-inflammatory environment despite the lack of STING signaling [22–24, 37]. This sterile inflammatory environment, frequently referred to as “inflammaging,” exacerbates metabolic imbalance by facilitating mitochondrial malfunction and oxidative stress in metabolic tissues [65–67]. Recent evidence suggests that cGAS deficiency promotes the molecular signs of aging, mitochondrial DNA leakage, and the senescence-associated secretory phenotype (SASP), which collectively contribute to systemic metabolic deregulation [20, 58, 61]. The age-related lipid phenotypes we noted—such as transient hypertriglyceridemia in young age succeeded by persistent hypercholesterolemia throughout life—may be considered as metabolic markers of accelerated cellular aging in the absence of cGAS [68].

Finally, the main advantage of this research is in its lifespan-centered experimental design, which investigates the metabolic effects of total cGAS deficiency under healthy, non-obese conditions. To our knowledge, this is the first report addressing the time aspect of cGAS–cGAMP interaction in metabolic control. Nonetheless, this study possesses intrinsic limitations. Cellular, molecular, protein, and enzymatic research were not conducted to directly validate the postulated mechanistic pathways, and the quantification of enzyme expression or activity, dsDNA receptors expression, NAD⁺ levels, or mitochondrial respiration beyond the limitations of the current design. Similarly, whereas DNA damage was not reassessed, it has been clearly established in cGAS-deficient mice that genomic instability induces aging-related inflammation and metabolic dysregulation [20, 58, 63, 69]. Subsequent research integrating transcriptomic, lipidomic, and metabolomic analyses will be essential to clarify the precise molecular pathways by which cGAS and cGAMP influence lipid metabolism, mitochondrial bioenergetics, and redox balance across various tissues and stages of development.

## Conclusions

Our results demonstrate that the absence of cGAS and cGAMP is associated with longitudinal and age-related metabolic changes. This phenotype is characterized by a biphasic adaptation, marked by transient metabolic responses such as decreased glucose levels and elevated triglyceride levels in early life, which subsequently progress into adipose hypertrophy and persisting hypercholesterolemia throughout the lifespan. Furthermore, we observed low-grade inflammation in the liver, diminished weight-bearing capacity, and increased body weight, all without affecting general locomotor activity. These findings reveal an unacknowledged metabolic role of the cGAS–cGAMP pathway that operates independently of STING, highlighting the pathway’s complexity and the necessity for careful interpretation in the context of therapeutic targeting.

## Data Availability

The datasets used and/or analyzed during the current study are available from the corresponding author upon reasonable request.
